# SERS active Ag–SiO_2_ nanoparticles obtained by laser ablation of silver in colloidal silica

**DOI:** 10.3762/bjnano.9.224

**Published:** 2018-09-06

**Authors:** Cristina Gellini, Francesco Muniz-Miranda, Alfonso Pedone, Maurizio Muniz-Miranda

**Affiliations:** 1 Department of Chemistry “Ugo Schiff”, University of Florence, Via della Lastruccia 3, 50019 Sesto Fiorentino, Italy; 2 Department of Chemical and Geological Sciences, University of Modena and Reggio Emilia, Via Campi 103, 41125 Modena, Italy; 3 Center for Molecular Modeling, Ghent University, Technologiepark 903, 9052 Zwijnaarde, Belgium

**Keywords:** 2,2’-bipyridine, DFT, laser ablation, silica, silver

## Abstract

Highly stable Ag–SiO_2_ nanoparticle composites were first obtained by laser ablation of a silver target in an aqueous colloidal dispersion of silica and examined by UV–vis absorption spectroscopy, transmission electron microscopy and Raman spectroscopy. The surface enhanced Raman scattering (SERS) activity of these nanocomposites was tested using 2,2’-bipyridine as a molecular reporter and excitation in the visible and near-IR spectral regions. The computational DFT approach provided evidence of ligand adsorption on positively charged adatoms of the silver nanostructured surface, in a very similar way to the metal/molecule interaction occurring in the corresponding Ag(I) coordination compound.

## Introduction

Silica nanoparticles in an aqueous suspension are known to be inert and dispersible – properties which can be quite useful in the preparation of colloidal nanocomposites with silver. For this reason, there are a significant number of studies in the literature on colloidal substrates formed with silica and silver, especially in recent years [1–14], which have found various applications in medicine, catalysis and environmental research. In this regard, it is important to remark that silica is an inert and optically transparent material.

The aim of the present work is to apply laser ablation to the fabrication of new materials for surface enhanced Raman scattering (SERS) [15–16], focusing on silver and silica nanoparticles in aqueous suspension. This research was undertaken for three main reasons. The first is that silver nanoparticles that adhere to silica can be stabilized in colloidal suspensions far better than in pure metal colloids, providing improved reproducibility of the SERS data. The second motivation is to exploit the adsorption capability of the colloidal silica for various organic ligands, for example glucose, formaldehyde, solvents as acetonitrile and benzene, and contaminants as polycyclic aromatic hydrocarbons (PAHs), polychlorinated biphenyls (PCBs), organochlorine pesticides, dioxins, nitroaromatic derivatives, and many others. All these compounds are inadequate to interact strongly with silver, but could be captured by silica, exhibiting suitable SERS enhancements by means of the vicinal presence of silver nanoparticles. Finally, the third reason is the use of laser ablation to obtain silver nanoparticles free of reagents and/or reaction products that could interfere in both ligand adsorption and SERS detection (as occurs when silver ions are chemically reduced), as proposed in several papers [17–23].

Here, we have ablated a silver target in a colloidal silica solution by nanosecond pulsed laser ablation and have verified the presence of Ag nanoparticles by UV–visible absorption spectroscopy and transmission electron microscopy (TEM). The SERS efficiency was tested by adsorption of 2,2'-bipyridine as a molecular reporter, whose SERS spectrum was interpreted by DFT, in order to gain information on the adsorbate and the interaction of the molecule with the metal surface. To our knowledge, this is the first investigation of this kind of preparation of a Ag–SiO_2_ colloidal nanocomposite and its application in SERS measurements.

## Experimental

The silver target was purchased from Aldrich (0.5 mm thickness, 99.9% purity), as well as 2,2’-bipyridine (purity 99%). Colloidal silica (Aldrich, Ludox TM-40, pH ≈9 at 25 °C, NaCl content: 0.03 wt %) was diluted with distilled water in order to have a sample with 10 wt % as SiO_2_ content.

Laser ablation of silver in colloidal silica was performed with the fundamental wavelength of a Q-switched Nd:YAG ns-pulsed laser (Quanta System G90-10: rep. rate 10 Hz, pulse width 10 ns). The laser pulse energy was set at 20 mJ/pulse (200 mW) with a laser spot of approximately 1 mm diameter and corresponding fluence of 2.5 J/cm^2^. The target plate was fixed at the bottom of a glass vessel filled with ≈4 mL of liquid (height above the target: 2 cm). The irradiation time of the silver target was about 10 min.

TEM samples were obtained by dropping a small amount of colloid onto carbon-coated copper grids and allowing it to evaporate. The images were recorded with a Philips CM12 at 120 kV.

UV–visible absorption spectra of the colloidal suspensions were obtained in the 200–800 nm region by using a Cary 5 Varian spectrophotometer.

SERS spectra were recorded using the 514.5 nm line of a Coherent Argon ion laser (power: 50 mW) and a Jobin-Yvon HG2S monochromator equipped with a cooled RCA-C31034A photomultiplier. Power density measurements were performed with a power meter (model 362; Scientech, Boulder, CO, USA) giving ≈5% accuracy in the 300–1000 nm spectral range. FT-SERS measurements were collected with a Fourier transform Raman spectrometer (Bruker Optics, Model MultiRam), equipped with a broad range quartz beamsplitter, an air-cooled Nd:YAG laser excitation source (1064 nm) and a Ge diode detector cooled with liquid nitrogen. The instrument provided a spectral range of 3600–50 cm^−1^ (Stokes shift). The experiments were performed in a 180° geometry, with 200 mW of laser power.

## Computational Details

All calculations were carried out using the GAUSSIAN 09 package [24]. Optimized geometries were obtained at the DFT level of theory with the Becke 3-parameter hybrid exchange functional combined with the Lee–Yang–Parr correlation functional B3LYP [25–26], along a mixed basis set made up of 6-311++G(d,p) for non-metal atoms and Lanl2DZ [27–29] for silver. All parameters were let free to relax and all calculations converged toward optimized geometries corresponding to energy minima, as revealed by the lack of negative values in the frequency calculation. A scaling factor of 0.98 for the calculated harmonic wavenumbers was employed, as usually performed in calculations at this level of theory [30–32]. The energies of formation for the bpy/silver complexes were calculated by considering the BSSE (Basis Set Superposition Error) correction.

## Results

**Figure 1:**
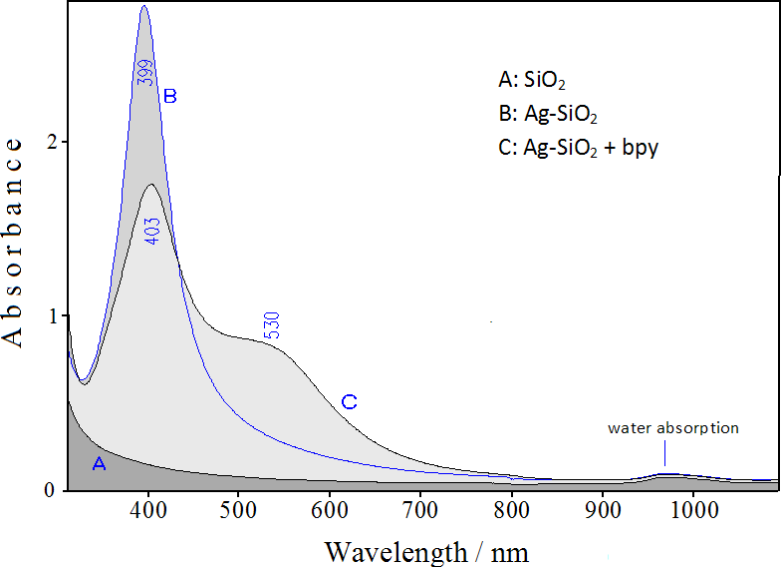
UV–visible absorption spectra of colloidal silica (A) and Ag-doped silica suspension before (B) and after addition of bpy (C).

The laser ablation of a silver target in an aqueous suspension of silica is illustrated in the scheme reported in Figure S1 of Supporting Information File 1. This procedure results in the emergence of the plasmonic band of nanometer-sized silver, observed in the absorption spectrum at 399 nm (Figure 1).

The shift of the maximum from 395 nm, as observed in Ag colloid obtained by ns-pulsed laser ablation [33], to 399 nm is consistent with the effect due to the higher refractive index of silica as compared to that of water [34]. This silver/silica colloidal suspension is very stable, as monitored in the UV–vis absorption. After more than 11 months after the preparation, the plasmonic band remains unchanged at the same wavelength and with the same bandwidth. Ten minutes of laser ablation are sufficient to obtain a plasmon absorbance more than 2.5 in a 10 mm cuvette. However, a much larger silver content could be obtained with longer ablation times without compromising the colloidal stability.

We have used an optimal pulse energy for the laser ablation of a silver target in colloidal silica, which allows ablating within a reasonable time and in a reproducible way, following the procedure previously reported in a recent paper [33]. Regarding the silica concentration, we have adopted the concentration used in the past (10 wt % SiO_2_) to obtain silica doped with silver [35]. This concentration is optimal for our purposes, that is, having a colloid with a high concentration of silica to exploit its adsorbent capacities, and limiting the background signal of silica. However, we have studied, during the laser ablation time, the growth of the plasmonic band of the silver nanoparticles in two silica suspensions, with 10 and 5 wt % SiO_2_ concentrations. The results appear quite similar, as shown in the Figure S2 of Supporting Information File 1.

**Figure 2:**
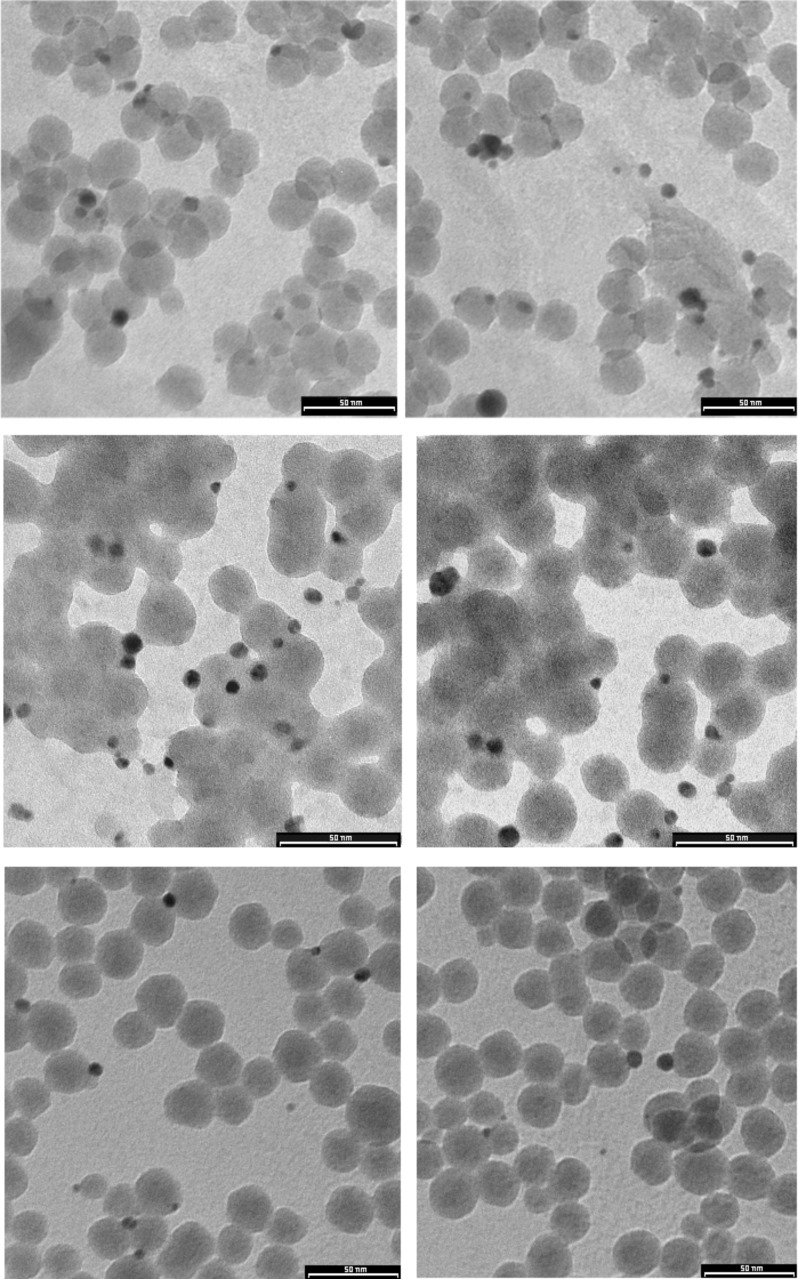
TEM micrographs of silica colloidal nanoparticles after laser ablation of silver.

The TEM images (Figure 2) show silver nanoparticles (5–10 nm) that are mostly located on the surface of the silica nanoparticles, which appear as spheres with uniform sizes (about 20 nm), or among them as bridges. There are no silver nanoparticles present that are not attached to silica nanoparticles. This could be due to the interaction of the laser-ablated silver nanoparticles, which have positive charges on the surface [36], with the negatively charged silanol groups of the silica surface. We have performed a study on the size distribution of the silver nanoparticles: more than half particles have a diameter between 4 and 6 nm, as shown in the graph reported in Supporting Information File 1 (Figure S3).

After addition of 2,2'-bipiridine (bpy), the plasmonic band decreases and moves to 403 nm (Figure 1), along with the growth of a secondary band with maximum around 530 nm. This evidence indicates that the ligand adsorption promotes the aggregation of the nanoparticles, which, however, does not result in the collapse of the colloidal suspension. In fact, the plasmonic bands remain unchanged, even after more than a week from the ligand addition.

This demonstrates that the colloidal suspension is well-stabilized by silica even in the presence of ligand molecules, so the performed SERS measurements are to be considered highly reproducible in time.

**Figure 3:**
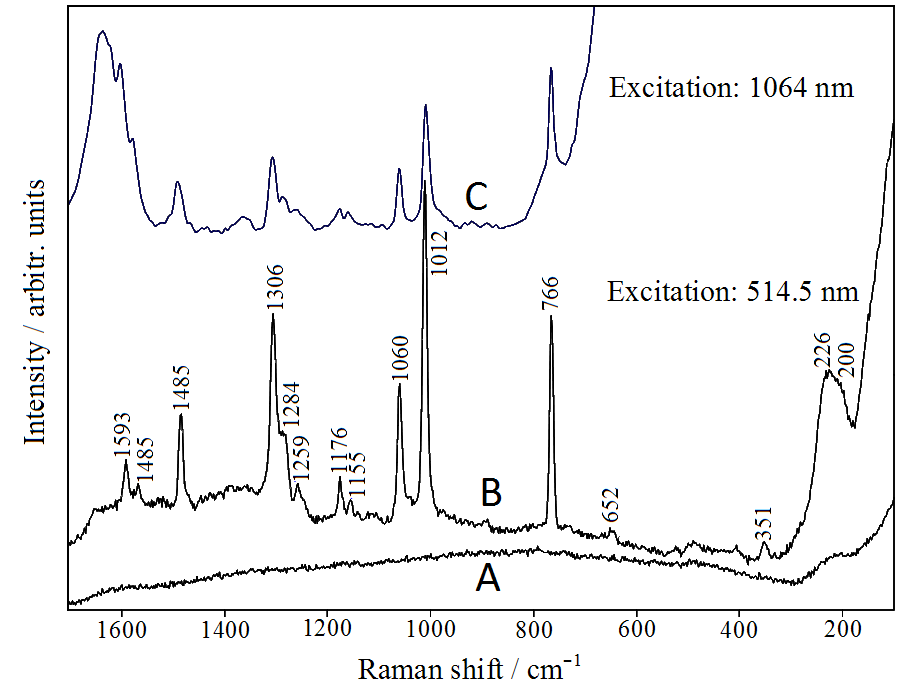
SERS spectra of bpy in Ag-doped silica colloid by excitation with the 514 nm (B) and 1064 nm (C) laser lines. The Raman spectrum of silica (514 nm) is also reported (A).

We have verified that our Ag–SiO_2_ nanocomposite is SERS active by addition of bpy as molecular reporter, as shown in Figure 3B by the strong Raman signals observed by laser excitation at 514.5 nm. The colloidal silica does not impair the observation of the ligand SERS bands because it does not have Raman bands that can interfere (see Figure 3A). The strong SERS response is due to the presence of chloride ions in the colloidal silica, which results in a marked increase of the SERS efficiency for silver nanoparticles obtained by laser ablation [37]. Moreover, SERS activity is observed also by laser excitation at 1064 nm, as shown by the FT-SERS spectrum reported in Figure 3C.

Also this evidence can be due to the presence of chloride anions in the colloidal silica suspension. It is known, in fact, that halides adsorbed on colloidal silver nanoparticles allow a SERS observation also for excitation in the near-IR spectral region [38–40]. This effect is attributable to the formation of charge-transfer complexes [41], which ensure SERS enhancement, even in the absence of an efficient resonance effect when the exciting radiation matches the plasmonic band. The observed SERS frequencies (see Figure 3B,C) correspond to those of bpy adsorbed on silver colloids [42–43], reported in Table 1. From a spectroscopic point of view, the silica/silver colloidal substrate presented in this work allows the SERS spectra to be completely comparable with those recorded in pure silver colloids, with regards to both frequency and intensity. In this regard, we present the SERS spectrum of 2,2’-bipyridine adsorbed on silver nanoparticles obtained by laser ablation in water in Figure S4 of the Supporting Information File 1. This suggests that even in the presence of silica, the ligand molecules are directly linked to the silver present in the colloidal suspension. To prove this indication and to understand the kind of molecule/metal interaction occurring, in addition to interpreting the observed SERS features, we have used the DFT computational approach, which provides important information on the adsorption of different molecules on SERS-active metals [44–47].

**Table 1:** Experimental and calculated frequencies (cm^−1^).

Raman Ag(I)(bpy)_2_NO_3_ [41,47]	Calc. Ag^+^(bpy) complex	Calc. Ag^0^(bpy) complex	SERS Ag/silica colloid	SERS Ag colloid [42]	SERS Ag colloid [41]

1590	1597	1584	1593	1596	1595
1571	1578	1576	1570	1566	1570
1485	1486	1482	1485	1485	1488
1429	1438	1431	–	1430	–
1304	1306	1309	1306	1307	1306
1282	1278	1274	1284	1284	1284
1259	1263	1258	1259	1260	1261
–	1185	1164	1176	1177	1177
1157	1167	1154	1155	1158	–
1062	1067	1055	1060	1063	1062
1012	1013	994	1012	1013	1012
815	824	822	–	–	–
768	762	767	766	767	766
655	654	633	652	655	649
622	631	616	–	624	618
356	345	377	351	353	356
237	239	240	226	–	–
–	194	–	200	–	–

Actually, the SERS bands observed in the Ag–SiO_2_ colloid appear quite similar to the Raman bands of the argentous coordination compound [42,48] Ag(I)(bpy)_2_, where the molecule in a cis conformation binds to a silver ion by means of both nitrogen atoms. To confirm that this interaction also occurs in our Ag–SiO_2_ colloid, we have performed DFT calculations by considering two possible complexes of bpy in a cis conformation, bonded to Ag^+^ ion or to Ag^0^ neutral atom. This computational approach is consistent with the chemical enhancement mechanism proposed by Otto et al. [49–50], which is based on the adatom model. In this model, the interaction of ligand molecules occurs with surface defects that are constituted by one or a few metal atoms, which can be considered almost isolated on the metal surface. The validity of the adatom approximation has been widely verified by DFT calculations of many adsorbed molecules and was able to satisfactorily reproduce the corresponding SERS spectra [51–53].

The DFT calculated frequencies of the two proposed complexes are shown in Table 1, along with the experimental ones. These calculations allow a comparison between observed and calculated frequencies, as well as the stability of the two proposed complexes by considering bpy–Ag bond distances and complexation energies. As one can note, the Ag^0^(bpy) complex model is unable to satisfactorily reproduce the SERS frequencies, especially those observed in the low-frequency region at 351 and 652 cm^−1^ and those relative to the most intense SERS band at 1012 cm^−1^, where the results are largely underestimated. The interaction of bpy with the Ag^0^ atom is very weak, as shown by both the scarce molecule → metal electronic charge-transfer and the long N–Ag bond distance, reported in Table 2. Moreover, the large difference between the complexation energies of the two proposed complexes indicates that the molecule by far prefers to bind to the positively charged silver atom than to a neutral one. The interaction of the molecule with Ag^+^ results in the formation of a complex characterized by a large charge transfer, more than half the electronic charge (Table 2). This model complex well reproduces the SERS frequencies, as well as those observed in the Raman spectrum of the Ag(I) coordination compound, as shown in Table 1. Also the two SERS bands observed in the low-frequency region at 226 and 200 cm^−1^ are satisfactorily reproduced by the Ag^+^(bpy) complex and attributed to the stretching modes of two N–metal bonds. The Mulliken charges calculated for the two complexes are reported in Table S1 of the Supporting Information File 1, while their optimized structures are shown in Figure S5. The N–Ag^+^ distances, as well as that of the inter-ring C–C bond, are quite similar to the corresponding experimental values obtained for bis(4,4',6,6'-tetramethyl-bpy)-Ag(I) coordination compound (N–Ag^+^ = 2.30 Å; C–C' = 1.499 Å), as reported in the literature [54].

**Table 2:** Calculated bpy → metal electronic charge-transfers, N–Ag distances in Ag^+^(bpy) and Ag^0^(bpy) complexes and relative complexation energies. e* = proton charge.

	Ag^+^(bpy)	Ag°(bpy)

bpy → metal charge-transfer	−0.524e*	−0.166e*
complexation energy (kcal/mol)	−78.73	−4.62
N–Ag distance (Å)	2.285	2.701

Such spectroscopic and structural similarities, as evidenced by DFT calculations, show that the interaction of the molecule with the colloidal silver closely resembles that occurring in the Ag(I)–bpy coordination compound. In fact, it is known that the presence of chloride ions in silver colloids promotes the formation of positively charged active sites on the surface of the silver nanoparticles [55–56], as experimental proved by XPS measurements, which show a significant amount of oxidized silver on the particle surface [57].

**Figure 4:**
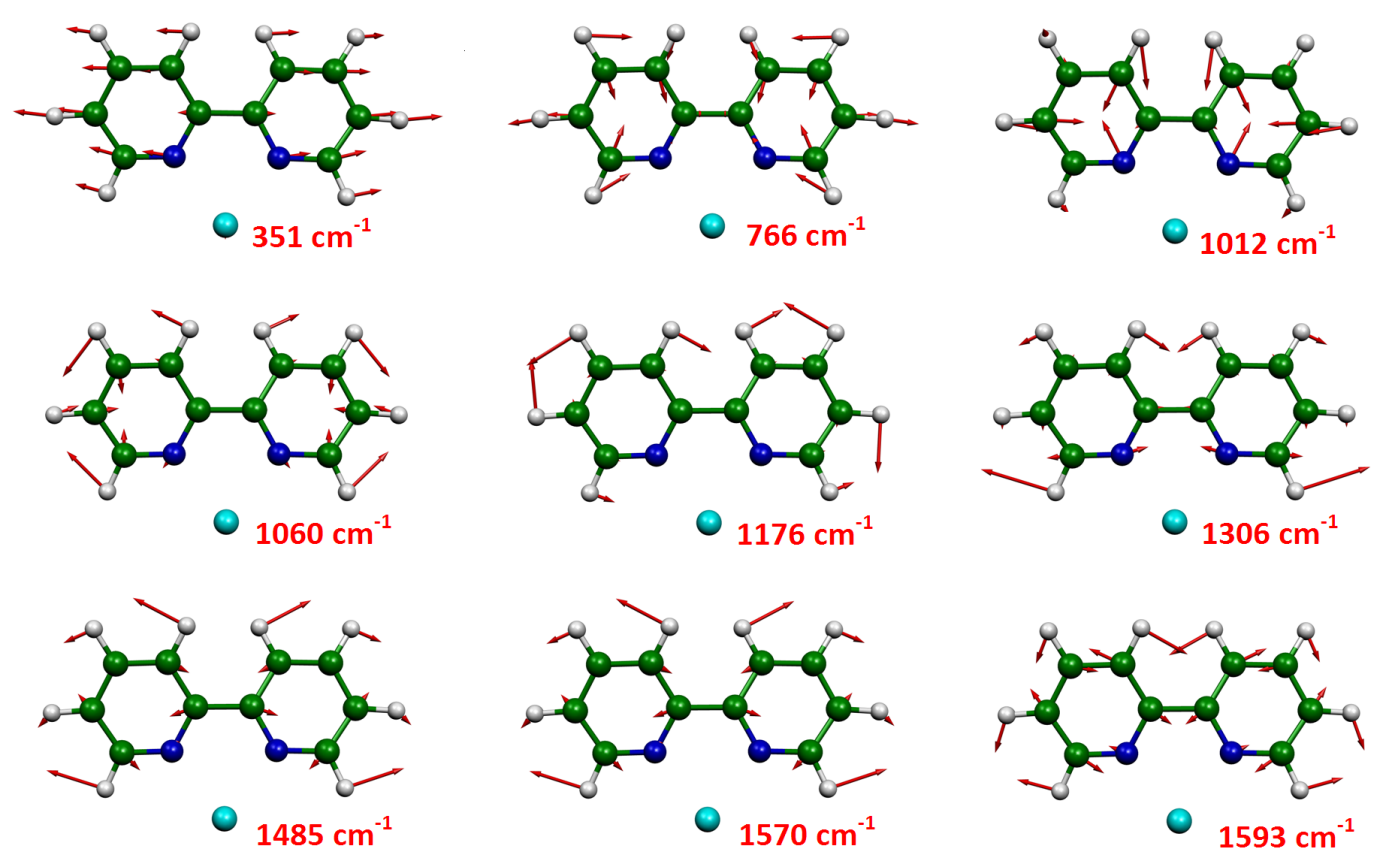
Calculated normal modes in terms of Cartesian displacements, corresponding to the prominent bands observed in the SERS spectra of bpy adsorbed on Ag-doped silica colloid.

Finally, Figure 4 shows the normal modes of the prominent SERS bands, which correspond to totally symmetric vibrations, except that at 1176 cm^−1^. The observed band at 351 cm^−1^ is mainly due to the inter-ring C–C’ stretching mode, and those at 766, 1012 and 1060 cm^−1^ are ring deformations. The peaks observed at 1176, 1303 and 1485 cm^−1^ are mainly attributable to ring H bending modes, while those at 1570 and 1593 cm^−1^ to C=C stretching modes.

## Conclusion

The results from this work are summarized in the following four points.

1. The silver/silica suspensions obtained by laser ablation are very stable, even after several months. The TEM images show that nanometer-sized silver is deposited on the surface of silica nanoparticles, instead of forming isolated Ag nanoparticles in the aqueous medium.
2. The Ag–SiO_2_ nanoparticles can effectively adsorb organic molecules such as 2,2'-bipyridine while maintaining their colloidal stability. The changes in the plasmonic bands observed in the UV–visible absorption spectrum after ligand addition indicate that the adsorbed molecules induce the aggregation of the nanoparticles, therefore favoring a good SERS activity. This is reinforced by the matching of the exciting radiation at 514.5 nm with the secondary plasmon band with maximum around 530 nm. The SERS spectrum of bpy in Ag–SiO_2_ colloid was found to be comparable with that obtained in pure silver colloid, in terms of frequency and intensity. This demonstrates that the interaction between silver and silica does not alter the SERS response, allowing all the spectroscopic applications provided by the SERS technique to be explored. However, the advantage of employing Ag–SiO_2_ nanoparticles lies in the exceptional capacity of colloidal silica to capture organic substances that are not able to interact directly with silver, but that can undergo SERS enhancement thanks to the vicinal presence of silver nanoparticles.
3. The Ag–SiO_2_ colloidal nanoparticles also show an efficient SERS response for organic ligands such as 2,2'-bipyridine at a laser excitation wavelength of 1064 nm. The possibility to observe FT-SERS signals increases the detection potential of this spectroscopic technique, as interference due to fluorescence does not usually occur by excitation in the NIR spectral region.
4. The computational DFT approach, in addition to allowing a correct assignment of the observed SERS bands, evidenced that 2,2'-bipyridine adsorbs on silver in a cis conformation linked to a positively charged active site (adatom) of the metal surface. This molecule/metal interaction closely resembles that occurring in the corresponding Ag(I) coordination compound. The strong molecule → metal charge-transfer, as evaluated by DFT calculations, can also explain the SERS effect obtained by NIR excitation, even if the excitation radiation does not match the plasmonic band of the silver nanoparticles.

In conclusion, a simple method is proposed to obtain Ag-doping of silica colloidal nanoparticles, avoiding complicated procedures and chemical reactants. These nanocomposites, which combine the adsorption properties of silica with the plasmonic properties of nanometer-sized silver, can be used for several purposes, for example, for the SERS detection of environmental contaminants such as POPs (persistent organic pollutants), as polycyclic aromatic hydrocarbons, polychlorinated biphenyls, organochlorine pesticides, dioxins, nitroaromatic derivatives, and many others. These molecules, which normally do not interact with silver, could be effectively adsorbed on silica nanoparticles.

## Supporting Information

File 1 Supporting Information.
