# Supplementary material for: SERS active Ag–SiO2 nanoparticles obtained by laser ablation of silver in colloidal silica
Source: Beilstein J Nanotechnol. 2018 Sep 6;9:2396–404. doi: 10.3762/bjnano.9.224 (PMC6142779; doi:10.3762/bjnano.9.224)
Supplement: File 1 — Supporting Information. [file Beilstein_J_Nanotechnol-09-2396-s001.pdf]

Supporting Information

for

SERS active $Ag-SiO_2$ nanoparticles obtained by laser ablation of silver in colloidal silica

Cristina Gellini[*1], Francesco Muniz-Miranda[2,3], Alfonso Pedone[2] and Maurizio Muniz-Miranda[1]

Address: [1]Department of Chemistry "Ugo Schiff", University of Florence, Via della Lastruccia 3, 50019 Sesto Fiorentino, Italy, [2]Department of Chemical and Geological Sciences, University of Modena and Reggio Emilia, Via Campi 103, 41125 Modena, Italy and [3]Center for Molecular Modeling, Ghent University, Technologiepark 903, 9052 Zwijnaarde, Belgium

Email: Cristina Gellini - cristina.gellini@unifi.it

*Corresponding author

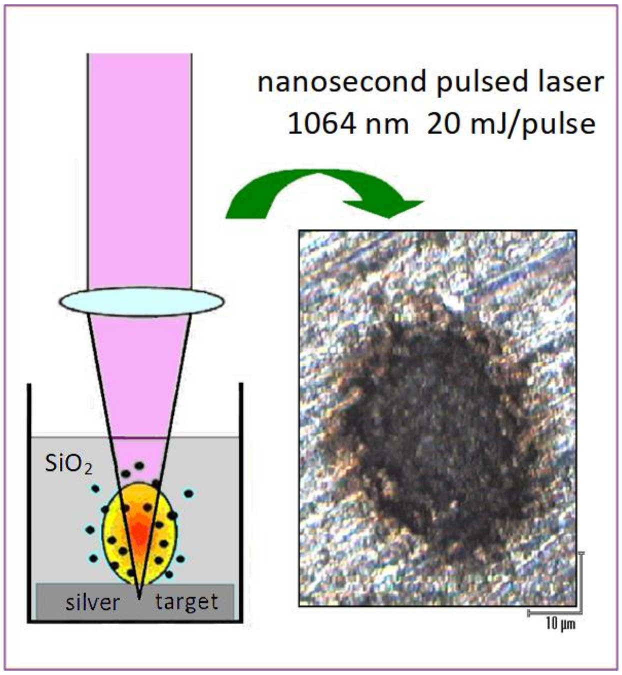

Figure S1: Scheme of the laser ablation procedure of a silver target in colloidal silica.

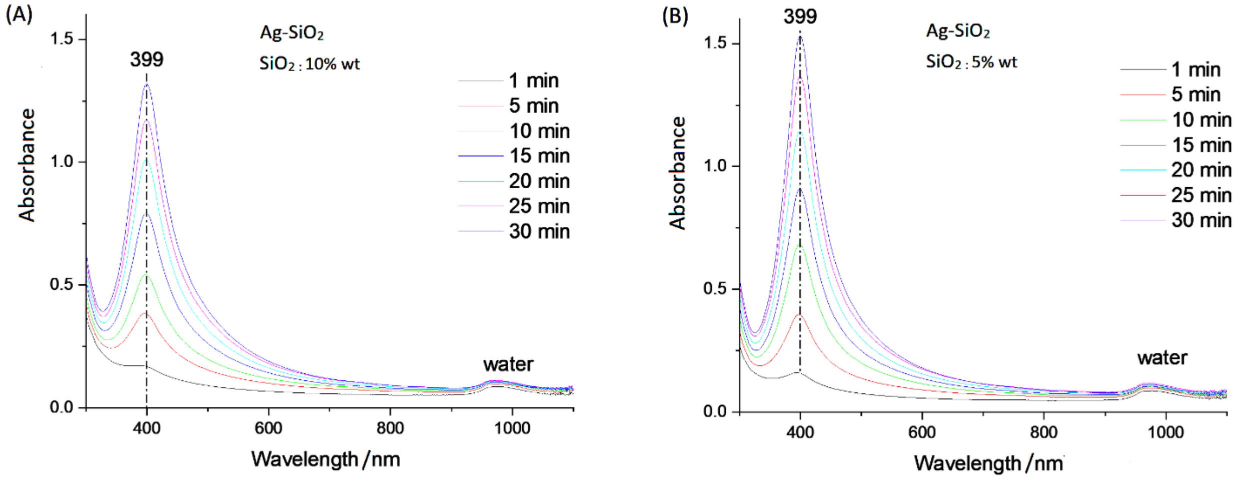

Figure S2: Time evolution of the plasmonic band of Ag-SiO_2 colloids with different silica content during laser ablation procedure.

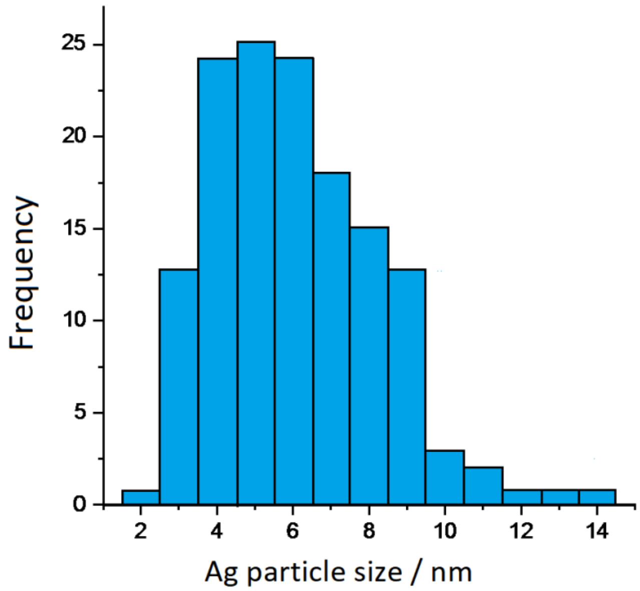

Figure S3: Size distribution of silver nanoparticles in the Ag-SiO_2 colloid.

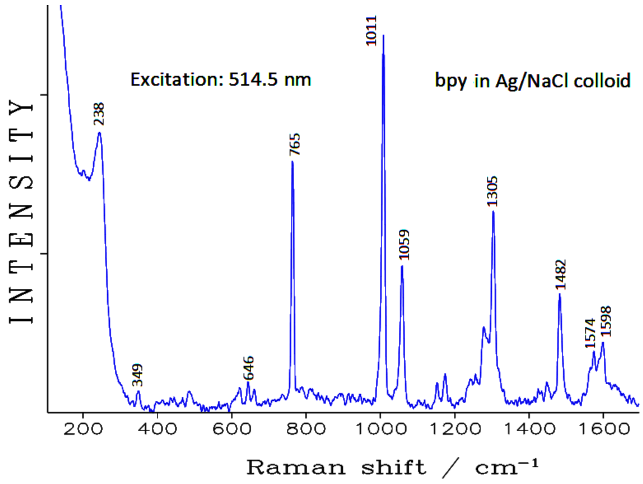

Figure S4: SERS of bpy in laser-ablated Ag colloid.

Table S1: Mulliken charges.[a]

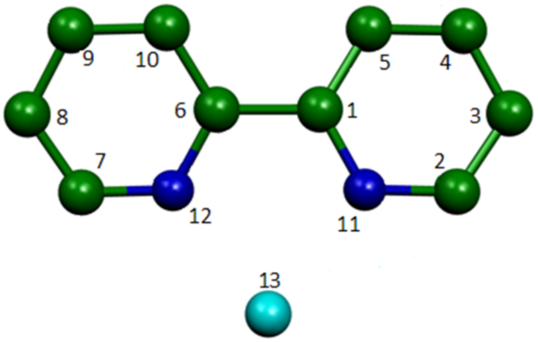

Atom	Ag^{+}(bpy)	Ag^{o}(bpy)
1 C	0.2101	0.1908
2 C	0.1620	0.1244
3 C	0.0728	0.0291
4 C	0.0833	0.0346
5 C	0.0276	-0.0347
6 C	0.2101	0.1908
7 C	0.1620	0.1244
8 C	0.0728	0.0291
9 C	0.0833	0.0346
10 C	0.0276	-0.0346
11 N	-0.2938	-0.2609
12 N	-0.2938	-0.2609
13 Ag	0.4758	-0.1664

[a]with hydrogens summed into heavy atoms.

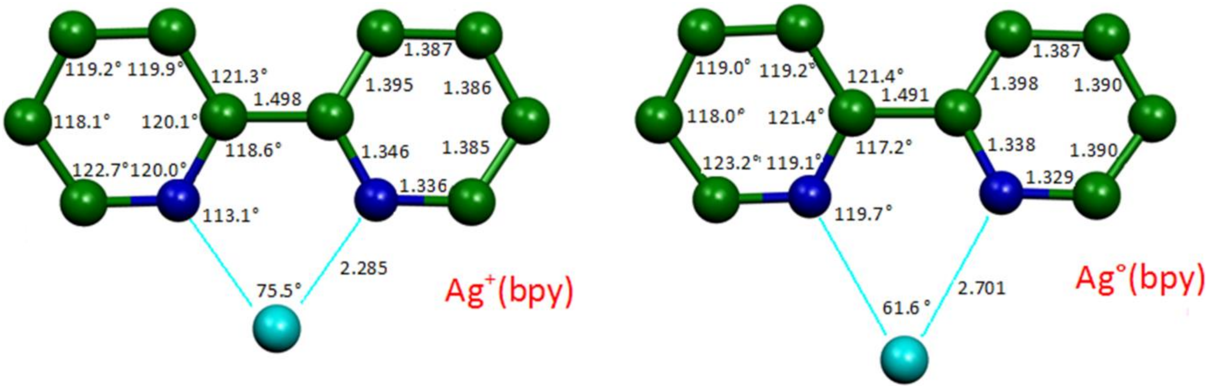

Figure S5: Structural parameters of the silver complexes.
